# Severity of Coronary Atherosclerosis and Risk of Diabetes Mellitus

**DOI:** 10.3390/jcm8071069

**Published:** 2019-07-21

**Authors:** Iginio Colaiori, Raffaele Izzo, Emanuele Barbato, Danilo Franco, Giuseppe Di Gioia, Antonio Rapacciuolo, Jozef Bartunek, Costantino Mancusi, Maria Angela Losi, Teresa Strisciuglio, Maria Virginia Manzi, Giovanni de Simone, Bruno Trimarco, Carmine Morisco

**Affiliations:** 1 Cardiovascular Research Center Aalst OLV Hospital, 9300 Aalst, Belgium; 2 Department of Advanced Biomedical Sciences, University of Naples Federico II, 80100 Napoli, Italy

**Keywords:** coronary artery disease, diabetes mellitus, atherosclerosis, coronary angiography

## Abstract

Background: Cardio-vascular target organ damage predicts the onset of type 2 diabetes mellitus (DM) in hypertensive patients. Whether an increased incidence of DM is also in relation to the severity of coronary atherosclerosis is unknown. Objective: We evaluated the onset of DM in relation to the extent and severity of coronary atherosclerosis, using the SYNTAX (Synergy between Percutaneous Coronary Intervention with Taxus and Cardiac Surgery) score (SS), in patients with stable angina or acute coronary syndromes, referred for coronary angiography (CA). Methods: Non-diabetic patients that underwent CA for the first time were included, and the SS was computed. Predictors of DM onset in low, medium, and high SSs were investigated. Results: Five hundred and seventy patients were included, and the mean SS was 6.3 ± 7.6. During a median follow-up of 79 months (interquartile range (IQR): 67–94), 74 patients (13%) developed DM. The risk of DM onset was significantly higher in the patients with a medium or high SS (hazard ratio (HR)—95% confidence interval (CI): 16 (4–61), *p* < 0.0001; and 30 (9–105), *p* < 0.0001, vs low SS, respectively), even after adjustment for obesity, history of hypertension, impaired fasting glucose, and cardiovascular therapy. Conclusions: The severity and extent of the coronary atherosclerosis, evaluated by the SS, is a strong and independent predictor of the development of DM in patients, referred to CA.

## 1. Introduction

Type 2 diabetes mellitus (DM) and cardiovascular (CV) disease are closely correlated. DM is associated with a two- to four-fold increased risk of coronary artery disease (CAD) and stroke [1]. On the other hand, CV diseases are the main causes of death and disability among patients with DM [2]. Furthermore, DM is associated with more extensive coronary atherosclerosis [3], and worse outcomes in acute coronary syndromes [4]. The association with coronary atherosclerosis spans from early stages of glucose intolerance to overt DM [5,6,7]. We previously demonstrated, in hypertensive patients, that uncontrolled blood pressure is associated with a two-fold increased risk of diabetes onset [8]. In addition, hypertension-mediated target organ damage (e.g., carotid atherosclerosis and left ventricular hypertrophy) is a significant predictor of DM onset, independently of the baseline metabolic profile, anti-hypertensive therapy, and other significant covariates [9]. Thus, a well-characterized phenotype of hypertensive patients, carrying features suggestive of a high atherosclerotic burden, places patients at a higher risk for developing DM during follow-up. However, at this time, there is no direct demonstration that atherosclerosis exposes patients to a higher risk of DM development with a dose-response pattern. Identifying patients with an increased risk of DM, in the setting of patients referred to Cat Labs for coronary angiography (CA), might be of paramount importance in terms of cardiovascular prevention [10]. Therefore, the early identification of individuals with established coronary artery disease at risk of type 2 DM could be further assessed for the severity of atherosclerosis, even before the clinical appearance of the disease, in order to promote the aggressive management of the metabolic profile in these patients.

Accordingly, we investigated whether the extension and severity of CAD diagnosed during CA might be predictive of the future onset of DM. 

## 2. Research Design and Methods

### 2.1. Patients

We screened all of the consecutive patients who underwent CA at the Cardiovascular Center Aalst (Belgium), between 1 January 2009 and 31 December 2009. The exclusion criteria were as follows: previous coronary angiogram, history of myocardial infarction or coronary artery bypass graft, or the diagnosis of pre-existing diabetes. All patients signed informed consent for CA and for data collection before the procedure. During the index hospital admission for CA, the blood pressure (BP), heart rate (HR), body mass index (BMI), fasting glucose, glycated hemoglobin (HbA1c), lipid profile, and kidney function were routinely assessed for each patient. All of the patients underwent trans-thoracic echocardiogram (TTE), analyzed offline by one expert reader under the supervision of a senior consultant, using dedicated work-stations (Echo-PAC Clinical Workstation Software, GE Healthcare, Horten, Norway).

The SYNTAX (Synergy between Percutaneous Coronary Intervention with Taxus and Cardiac Surgery) score (SS) was calculated for all of the patients by two interventional cardiologists blinded to the baseline clinical characteristics, procedural data, and clinical outcomes. Each coronary lesion with more than a 50% diameter stenosis in vessels of at least 1.5 mm, by visual estimation, was scored separately using the SS algorithm from the related website [11]. To assess the intra-observer reproducibility, angiograms were re-analyzed by the same interventional cardiologist eight weeks after the first analysis. The investigator remained blinded to the results of the first analysis. After the first admission for CA (index procedure), patients were assessed for incidence of type 2 diabetes in all of the subsequent follow-up visits or laboratory exams at the Cardiovascular Center of Aalst. To standardize the follow-up (FU), we analyzed the ambulatory visits and laboratory data every 6 months, until the last FU. 

### 2.2. Primary Endpoint

The primary endpoint of the study was the incidence of type 2 DM after index hospitalization. Diabetes was defined according to the 2018 American Diabetes Association (ADA) criteria, as follows: fasting plasma glucose of 126 mg/dL (7.0 m mol/L) or hemoglobin A1C of 6.5% (48 mmol/mol) [12]. To accurately date the first diagnosis of diabetes, we carefully checked on the initiation of anti-diabetic therapies, and on the laboratory data at the occasion of the outpatient clinic visit. The onset of diabetes was adjudicated based on the earliest evidence of ADA criteria at the follow-up.

### 2.3. Measurements and Definitions

Obesity was defined as a BMI of 30 kg/m^2^. According to the ADA criteria, impaired fasting glucose (IFG) was considered when the fasting plasma glucose was between 101 and 125 mg/dL. The systolic and diastolic blood pressure (BP) was measured by standard aneroid sphygmomanometer after 5 min rest in the supine position. Three BP measurements were obtained in the sitting position, at 2 min intervals. The averages of these measurements were used for the analysis. Hypertension is defined as office systolic blood pressure (SBP) values of 140 mmHg, and/or diastolic BP (DBP) values of 90 mmHg, according to the European Society of Cardiology (ESC) guidelines [13]. We defined peripheral vascular disease (PVD) as disease documented by a vascular imaging study (including a Computed tomography (CT) scan, ultrasound, peripheral angiography, and magnetic resonance imaging (MRI) that was significant enough for the patient to be referred for elective vascular surgery or percutaneous intervention.

### 2.4. Statistical Analysis

The data were analyzed using IBM SPSS Statistics (version 25.0; SPSS, IBM, Armonk, NY, US), and expressed as mean ± 1 standard deviation (SD). The variables that were not normally distributed were log-transformed. The study population was divided into quartiles of SS. For the exploratory statistics, we considered the two lowest quartiles as low-risk SS (group 1); the third one, corresponding to the median of distribution, as moderate-risk SS (group 2); and the highest one, corresponding to the 75th percentile of the distribution, as high-risk SS (group 3). Analysis of variance (ANOVA) was used to compare the baseline characteristics of the three groups of patients according to SS. Under the assumption of increasing abnormalities from group 1 to group 3, polynomial linear and quadratic contrasts were used to estimate the trend. The χ2 distribution was used to compare the categorical variables, with the Monte Carlo simulation in order to obtain exact *p*-values. 

The incidence of diabetes in relation to the three groups of SS was assessed using three models of Cox regression analysis, as follows: (a) in the first model, age, gender, and metabolic profile were included; (b) in the second model, the echo parameters and classes of drugs were included; (c) in the third model, the dosages (low vs high dosages) of the statins (i.e., patients on rosuvastatine ≥20 mg/die or atorvastatine ≥40 mg/die were considered as high dosage) were included. A two-tailed *p*-value of <0.05 was used to reject the null hypothesis.

## 3. Results

### 3.1. Patients

Within the study period, 570 non-diabetic (mean age 65 ± 10 years; 69% males) patients fulfilled the inclusion and exclusion criteria, and were included in this analysis. Table 1 shows the demographic and clinical characteristics of the patients stratified by SS. In the patient population, the included SSs were as follows: SS low, 0 to ≤4; SS medium, >4 to ≤10; and SS high, >10. The patients in the high SS were older, and showed a lower heart rate and left ventricular ejection fraction. The basal fasting plasma glucose was found to be progressively higher among the patients with medium and high SS, while the lipid profile was more favorable in the medium SS. Table 2 shows the medical therapy at discharge from the index hospitalization. All four classes of medications considered in the analysis were prescribed more frequently in patients with a higher SS.

### 3.2. Follow-Up

**Figure 1:**
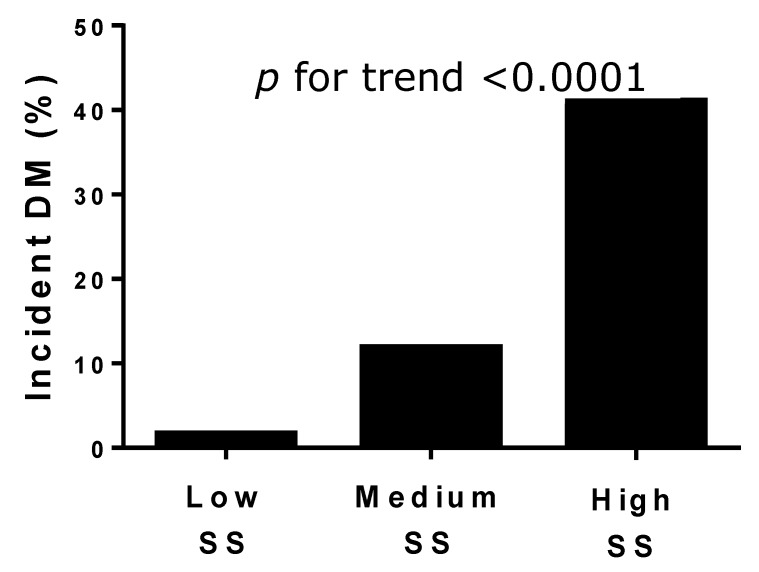
Rate of type 2 diabetes mellitus (DM) in the three SYNTAX score (SS) levels.

**Figure 2:**
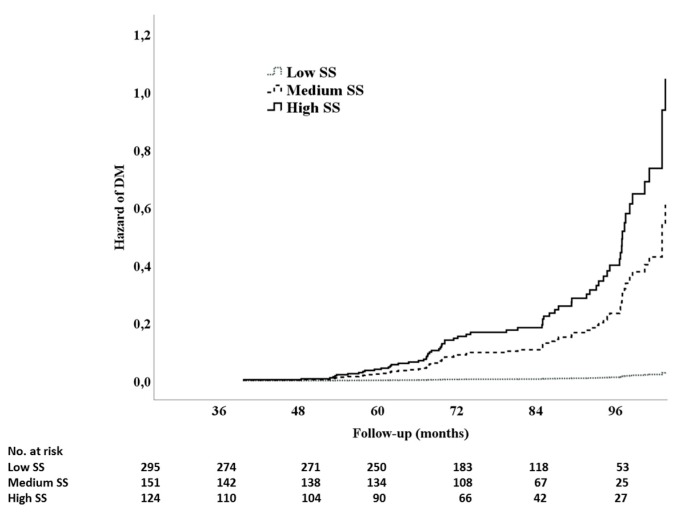
Cox regression time to event analysis for type 2 diabetes mellitus onset, in the function of the three SS levels.

During a median follow-up of 79 months (interquartile range (IQR): 67–94), 74 (13%) patients developed DM. The incidence of diabetes was significantly higher in the patients with a high SS (41%), than in patients with a medium (12%) and low SS (2%; *p* for trend < 0.0001; Figure 1). In the Cox regression analysis (Table 3), the predictors of DM onset during follow-up had higher baseline values of fasting plasma glucose (HR = 1.043; (95% CI 1.011–1.075); *p* < 0.008), medium SS (HR = 6.630; (95% CI 2.394–18.358); *p* < 0.0001), and high SS (HR = 14.789; (95% CI 5.796–37.737); *p* < 0.0001). The three curves (Figure 2) started to separate after 48–52 months from the index hospitalization, and further diverged after five years of follow-up. In the second Cox regression analysis performed by including the ejection fraction, heart rate, and the drugs prescribed (Table 4), the predictors of the new onset of DM were as follows: use of statins (HR = 6.953; (95% CI 1.618–29.880); *p* = 0.009), an anti-renin angiotensin system (RAS; HR = 3.338; (95% CI 1.917–5.812); *p* = 0.0001), medium SS (HR = 6.022; (95% CI 1.714–21.158); *p* = 0.005), and high SS (HR = 13.140; (95% CI 3.857–44.738); *p* < 0.0001) was further reinforced (Table 4). In the last model (Table 5), including the dosage of statins, the independent predictors of DM were a low dose of statins (HR = 8.631; (95% CI 2.017–36.931); *p* = 0.004), a high dose of statins (HR = 8.158; (95% CI 1.764–37.728); *p* = 0.007), anti-RAS (HR = 3.637; (95% CI 2.151–6.6.151); *p* < 0.0001), medium SS (HR = 3.505; (95% CI 1.274–9.644); *p* = 0.015), and high SS (HR = 8.906; (95% CI 3.404–23.296); *p* = 0.0001).

In patients who developed DM, no significant differences were detected in the incidence of major adverse cardiovascular events (i.e., death, myocardial infarction, or coronary revascularization) among the three SS subgroups (*p* = 0.418).

## 4. Discussion

In non-diabetic patients, our study demonstrates, for the first time, a significant association between the severity and extent of coronary atherosclerosis, assessed by the SS, with the future development of type 2 DM. Compared with patients with low SS, those with medium or high values exhibited an 8-fold and 10-fold higher risk for developing type 2 DM during follow-up, an association that was independent of potential confounders, including initial metabolic profile, age, anthropometric and hemodynamic characteristics, and medications. 

### Vascular and Metabolic Disease

Our results parallel previous studies challenging the paradigm that type 2 DM normally precedes and portends to a higher risk of developing vascular atherosclerosis [14,15], although they provide direct evidence of this reverse temporal relation. Our results are consistent with findings in patients with essential hypertension, in whom both resistant hypertension [8] and left ventricular hypertrophy [9] precede the onset of type 2 DM. Our findings extend to patients with coronary artery disease (CAD), the evidence of this reverse temporal relation. We hypothesize that a possible vicious circle might be at the basis of this phenomenon. It is reasonable to speculate that the mechanisms relating atherosclerosis to the later onset of DM might be related to insulin resistance, rather than directly to a hyperglycaemic state. However, as the present study was not designed to explore the pathogenic mechanisms underlying the association between SS and the development of diabetes, the insulin sensitivity in our study population remained unexplored. Insulin resistance is a common pathogenic background for both DM and atherosclerosis [16,17]. Insulin resistance plays a mechanistic role, along with other risk factors, in the development of vascular damage and the occurrence of cardiovascular events (four) [18,19]. In particular, insulin resistance-mediated endothelial dysfunction (an early step of atherosclerosis) [20] has been proposed as a pathogenic mechanism of DM [21]. In turn, the presence of an extensive vascular atherosclerosis might facilitate the onset and maintenance of the insulin resistance state. This vicious circle can well explain how CV damage, instead of being a consequence, could progress, together with the development of DM.

Our results cannot exclude the possibility that sub-clinical DM was already present at the time of the index CA in patients who clinically manifested the disease during follow-up. As matter of fact, endothelial dysfunction precedes and predicts incident diabetes, supporting the hypothesis that vascular disease might precede pancreatic beta-cell failure, determining the shift from insulin resistance to diabetes [22]. Moreover, endothelial dysfunction and impaired nitric oxide-mediated vasodilatation have also been suggested to directly lead to reduced insulin delivery to skeletal muscles, resulting in peripheral insulin resistance and hyper-glycaemia [23].

It is of note that patients with medium or high SS manifested type 2 DM after a subclinical phase of nearly four years from the index CA. As SS was evaluated cross-sectionally, this study does not clarify whether the association between the magnitude of coronary atherosclerosis and the development of type 2 DM is a time-dependent or atherosclerotic-dependent phenomenon. However, the latter seems to play an important role, considering that the rate of DM increases with increasing the SS category (Figure 1).

The evidence that patients with more than a low SS have a much higher risk of incident type 2 DM is of clinical importance. It implies that these patients, in addition to their regular cardiovascular follow-up, require tailored management in terms of metabolic risk, including attention to pharmacological therapy. In particular, it has been demonstrated that a high-dose regimen with statins was associated with an increased risk of new-onset DM [24,25]. In keeping with these findings, we found that statin intake was significantly associated with more complex coronary artery disease, and was predictive of an increased rate of DM.

In terms of the choice of revascularization strategies, the knowledge of the higher risk of development of type 2 diabetes in patients with more than a low SS might favor surgical over percutaneous interventions, after taking into account the anatomical features of the CAD and the clinical conditions of the patient [26]. 

## 5. Conclusions

Our study demonstrates that the extension and severity of coronary atherosclerosis is a strong predictor of development of type 2 DM. This finding advocates the need for developing dedicated management strategies in patients with severe coronary artery disease, taking into account the risk of late metabolic impairment following the index coronary intervention.

## Figures and Tables

**Table 1:** Baseline demographic and clinical characteristics of participants stratified by SYNTAX score (SS).

	Low SS (*n* = 295)	Medium SS (*n* = 151)	High SS (*n* = 124)	*p* (for trend)
Age (years)	65.5 ± 10.4	63.9 ± 10.5	67.1 ± 10.3	0.045
Sex (male/female %)	68.8/31.2	72.8/27.2	74.2/25.8	NS
Smokers (%)	47.5	53.0	48.4	NS
Hypertensives (%)	67.5	76.8	75.8	NS
BMI (Kg/m^2^)	27.1 ± 4.5	27.2 ± 4.3	27.3 ± 5.4	NS
Systolic BP (mmHg)	133.3 ± 22.0	128.8 ± 17.1	133.2 ± 19.4	NS
Diastolic BP (mmHg)	69.5 ± 11.4	69.3 ± 10.6	69.3 ± 11.3	NS
HR (bpm)	67.7 ± 12.2	66.9 ± 11.9	64.6 ± 8.7	0.048
Ejection Fraction (%)	67.9 ± 16.0	64.9 ± 14.0	61.6 ± 15.4	0.001
Fasting plasma glucose (mg/dL)	83.8 ± 13.2	85.6 ± 13.4	94.7 ± 13.5	0.0001
HbA1c (mmol/mol)	40.5 ± 8.7	40.4 ± 7.3	41.0 ± 7.5	NS
Total cholesterol (mg/dL)	172.6 ± 44.2	154.4 ± 36.6	156.0 ± 41.6	0.0001
LDL cholesterol (mg/dL)	91.8 ± 39.0	77.8 ± 31.4	81.1 ± 34.7	0.001
HDL cholesterol (mg/dL)	52.2 ± 16.8	51.0 ± 17.9	46.4 ± 14.5	0.01
Triacylglycerols (mg/dL)	147.3 ± 83.0	124.4 ± 67.4	146.1 ± 87.2	0.044
GFR_EPI_ (mL/min/1.73 m^2^)	64.7 ± 28.3	70.5 ± 25.6	64.3 ± 24.8	NS
CRP (mg/L)	27.7 ± 77.1	15.9 ± 34.0	28.7 ± 67.5	NS
IFG (%)	12.5	15.2	36.3	0.0001

BMI = body mass index; BP = blood pressure; HR = heart rate; HbA1C = hemoglobin glycated A1C; LDL = low density lipoprotein; HDL = high density lipoprotein; GFR = glomerular filtration rate; CRP = C-reactive protein; IFG = impaired fasting glucose; NS = not significant.

**Table 2:** Classes of drugs prescribed at the discharge.

	Low SS (*n* = 295)	Medium SS (*n* = 151)	High SS (*n* = 124)	*p*
CCB (%)	2.7	8.6	14.5	<0.0001
β-blockers (%)	20.0	37.7	42.7	<0.0001
Statins (%)	39.0	76.8	83.9	<0.0001
Statins low dose (%)	32.5	58.3	64.5	<0.0001
Statins high dose (%)	6.5	18.5	19.4	<0.0001
Anti-RAS (%)	29.5	27.2	46.8	0.001

SS = SYNTAX score; CCB = calcium channel blockers; RAS = renin angiotensin system.

**Table 3:** Results of Cox regression, including the age, gender, and metabolic profile.

Predictors	Sig.	HR	95.0% CI	
			Lower	Upper
Sex (1 male/2 female)	0.583	1.163	0.679	1.993
Age (years)	0.085	1.026	0.996	1.056
Fasting plasma glucose (mg/dL)	0.008	1.043	1.011	1.075
LDL Cholesterol (mg/dL)	0.323	1.130	0.535	2.385
IFG (y)	0.749	7.865	2.817	21.959
Medium SS	0.000	6.630	2.304	18.358
High SS	0.000	14.789	5.796	37.737

Abbreviations as in Table 1. CI = confidence interval.

**Table 4:** Results of Cox regression including ejection fraction, heart rate, and classes of drugs.

Predictors	Sig	HR	95.0% CI	
			Lower	Upper
Ejection Fraction (%)	0.698	0.996	0.978	1.015
HR (bpm)	0.128	1.021	0.994	1.048
CCB (*y*)	0.549	0.215	0.643	2.296
Β-blockers (*y*)	0.416	1.223	0.753	1.986
Statins (*y*)	0.009	6.953	1.618	29.880
Anti RAS (*y*)	0.000	3.338	1.917	5.812
Medium SS	0.005	6.022	1.714	21.158
High SS	0.000	13.140	3.857	44.768

Abbreviations as in Table 1 and Table 2.

**Table 5:** Results of the COX regression, including the classes of drugs and statins dosage.

Predictors	Sig	HR	95.0% CI	
			Lower	Upper
CCB (y)	0.387	1.314	0.708	2.440
Β-blockers (y)	0.390	1.238	0.761	2.013
Statins low dose (y)	0.004	8.631	2.017	36.931
Statins high dose (y)	0.007	8.158	1.764	37.728
Anti RAS (y)	0.0001	3.637	2.151	6.151
Medium SS	0.015	3.505	1.274	9.644
High SS	0.0001	8.906	3.404	23.296

Abbreviations as in Table 1 and Table 2.
